# Sensory Modality Shapes the Representational Structure of Emotion Recognition †

**DOI:** 10.3390/brainsci16090935

**Published:** 2026-08-31

**Authors:** Marilyn Mugure Chege, Fan Yang, Saige Lovell, Anqi Hu, Leila Moradkhani, Renee Leow-Guerville, Bobby Stojanoski, Ryan A. Stevenson

**Affiliations:** 1 Department of Psychology, University of Western Ontario, London, ON N6A 3K7, Canadaahu233@uwo.ca (A.H.); lmoradkh@uwo.ca (L.M.); ryan.stevenson@uwo.ca (R.A.S.); 2 Western Institute for Neuroscience, University of Western Ontario, London, ON N6A 3K7, Canada; fyang334@uwo.ca (F.Y.);; 3 Centre for Brain and Mind, University of Western Ontario, London, ON N6A 3K7, Canada; 4 Program in Neuroscience, Schulich School of Medicine and Dentistry, University of Western Ontario, London, ON N6A 5C1, Canada; 5 Department of Psychology, Ontario Tech University, Oshawa, ON L1G 0C5, Canada

**Keywords:** emotion recognition, representational similarity analysis, multidimensional scaling, hierarchical clustering, multisensory integration

## Abstract

Background: Emotion recognition is a fundamental aspect of social communication, yet most research has focused on visual emotion expressions, leaving it less understood how auditory and audiovisual cues shape perceptual organization. The present study used representational similarity analysis (RSA) to examine how the structure of emotion recognition differs across modalities. Methods: Participants completed an emotion recognition task involving 14 emotions presented in auditory, visual, and audiovisual formats. For each modality, a confusion matrix was constructed from the distribution of emotion labels participants chose for each target emotion. To separate confusion structure from overall accuracy, the primary analyses used error-only profiles (the off-diagonal responses, excluding correct answers), which were converted into representational dissimilarity matrices (RDMs). Structure was characterized with hierarchical clustering and multidimensional scaling (MDS) of the same RDM, with the number of clusters selected by silhouette and with reliability and stability assessed by resampling. Results: Recognition accuracy was lower for auditory than for visual and audiovisual presentations, which did not differ. Audiovisual and visual structures were strongly correlated and organized by valence, whereas the auditory structure diverged from both and showed no valence organization. Audiovisual structure corresponded most closely to a modality-general reference, followed by visual and then auditory. In the auditory modality, emotions of similar arousal but opposite valence were more confusable. These findings indicate that audiovisual and visual emotion perception share a largely valence-based organization, whereas auditory perception is organized along partly different lines, underscoring the value of examining the representational structure of emotion recognition beyond accuracy.

## 1. Introduction

Emotion categorization refers to how we perceive and classify emotional states. The study of emotion categorization has roots in both discrete and dimensional models, each offering distinct frameworks for understanding emotional experiences. Well-established theories categorize emotions either as discrete, based on facial and vocal features [1,2,3,4], or as dimensional, defined by underlying components such as arousal and valence [5,6]. Discrete models emphasize the universality of certain facial expressions and propose six basic emotions, including happiness, sadness, anger, fear, disgust, and surprise. Despite slight variations across models, these basic emotions are expressed and recognized with consistent facial patterns across cultures [1]. Each emotion is associated with specific expressive features, suggesting biologically ingrained response patterns. In contrast, dimensional models conceptualize emotions as positions along a continuous, multidimensional space where the axes are arousal (activation–deactivation) and valence (positive–negative). According to this perspective, emotional experiences are represented as gradients rather than discrete states [6].

**Figure 1:**
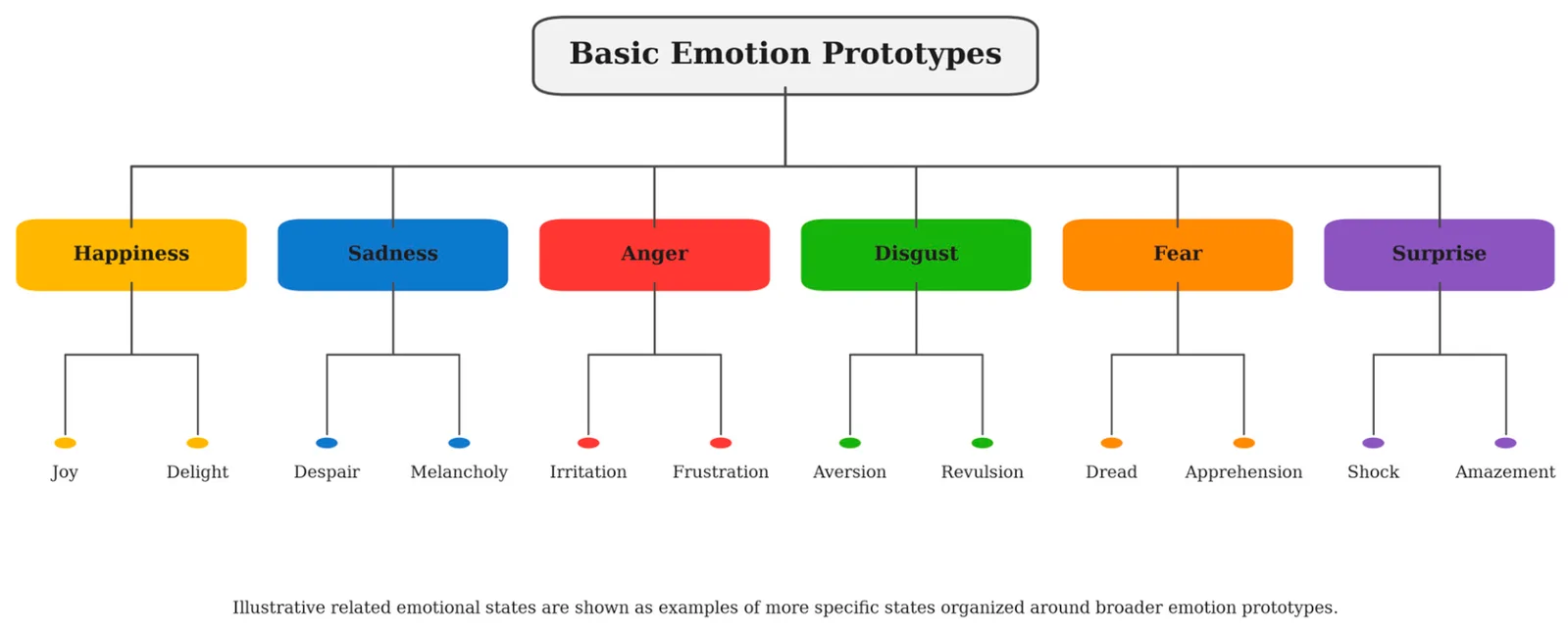
Hierarchical organization of emotion concepts by perceptual similarity. Basic emotion prototypes are shown as higher-level categories, with more specific emotional states represented as related examples within each prototype family. The diagram is intended as a conceptual illustration of similarity-based organization rather than an empirical clustering solution from the present study.

When considering perceptual similarity, basic and dimensional theories of emotions are not mutually exclusive. Perceptual similarity has been widely used to study emotion categorization, offering a different framework for understanding how emotions are grouped based on shared features. When similarity ratings are examined, emotions often organize into hierarchical structures, with basic emotions functioning as core prototypes that anchor more nuanced emotional states, which may vary along dimensional components [7]. Emotions that occur in similar contexts or have similar expressive features tend to cluster together. For instance, fear-related states, such as dread and apprehension, typically arise in response to perceived threat and involve expressions like trembling or nervousness. These features help distinguish emotions in the fear cluster from those associated with other categories. Similarly, a different emotion wheel model developed using similarity ratings organizes emotions in a circular arrangement based on intensity and valence, with basic emotions serving as the building blocks for more complex emotional experiences [8]. Fear, terror, and apprehension appear near one another in this framework as well, reflecting shared affective and perceptual properties. Across both models, perceptual similarity serves as a key organizing principle. Figure 1 illustrates the hierarchical organization of emotions, in which broad emotion categories are represented by core prototypes and more nuanced emotional states are grouped according to their perceptual similarity. 

Emotion perception is most often studied in the visual modality, where facial expressions serve as the primary cues for emotion recognition. Visual cues often convey emotional information through distinct and static facial configurations, resulting in relatively clear categorical distinctions. Emotions can also be recognized using only auditory cues like pitch, loudness, tempo, and timbre [2,9,10]. Vocal cues are dynamic, unfold over time, and are often expressed via both intensity and pitch, which results in greater ambiguity between emotions with similar arousal levels, such as fear and anger. Prior research has shown that emotions, especially basic emotions, exhibit cross-cultural similarity in auditory expression [11]. Although emotions can be recognized from vocal or facial expressions alone [1,12], emotions are typically conveyed through facial and vocal cues simultaneously, providing a richer representation of the emotion. Indeed, studies report that audiovisual presentations yield greater recognition compared to single-modality presentations [13,14].

Despite modality-specific differences in emotion recognition accuracy, little work has compared perceptual similarity across sensory modalities, but has focused on unimodal recognition, and it is unclear if emotional categories are perceived/organized similarly using visual, auditory, or audiovisual cues. The present study (1) explores if perceived similarity and clustering patterns differ across modalities; and (2) provides novel similarity metrics and clustering on a highly used emotional stimulus set in auditory, visual, and audiovisual modalities. Understanding how emotional similarity patterns differ by modality will provide insight into whether emotional categories are perceived as modality-dependent or as part of a unified, cross-modal structure. We predict that both similarity and clustering patterns will vary depending on the modality through which the emotion is presented, as each modality may emphasize different expressive features that affect how emotions are perceived and clustered.

## 2. Method

### 2.1. Participants

A total of 131 undergraduate students were recruited from the Department of Psychology research participant pool at Western University. Participants were retained for analysis if they provided valid responses on at least 95% of scored trials in each modality. All participants were aged between 17 and 22 years. The sample included 37 males, 92 females, and 2 individuals who preferred not to disclose their gender. All participants provided written informed consent, and all protocols were approved by the University of Western Ontario Non-Medical Research Ethics Board (REB number: 124088).

### 2.2. Stimuli

Emotional expression stimuli were selected from the Geneva Multimodal Emotion Portrayals Database [13]. This database features 1- to 3-s-long media clips of ten professional actors expressing 14 emotions, both positive and negative, using pseudo-utterances. The emotions included anger, irritation, disgust, sadness, despair, fear, anxiety, surprise, interest, relief, pleasure, amusement, joy, and pride. Each emotion is available in three formats: audio-only, visual-only, and audiovisual. The clips incorporate facial expressions, vocal cues, and body language to provide an ecologically valid measure of emotion recognition ability.

For each emotion, six portrayals by different actors were chosen for each modality following inspection of the available stimuli by the research team, resulting in 18 clips per emotion. To include all selected stimuli while minimizing participant fatigue, the materials were divided in half, with each set containing three non-overlapping clips per emotion per modality. A total of 58 participants completed the first half of the set, while 73 completed the second half. Each version contained three actor portrayals per emotion in each modality. Thus, each participant completed 42 trials per modality (14 emotions × 3 portrayals), 126 trials in total. The number of scorable responses contributing to each emotion within each modality is reported in Table S1. For a given emotion-by-modality combination, the actor portrayals used in one version were not used for that same combination in the other version. Actor identity was not systematically matched across modalities. Since the stimuli were drawn from a finite actor pool, the same actor could portray the same emotion in more than one modality.

### 2.3. Procedure

**Figure 2:**
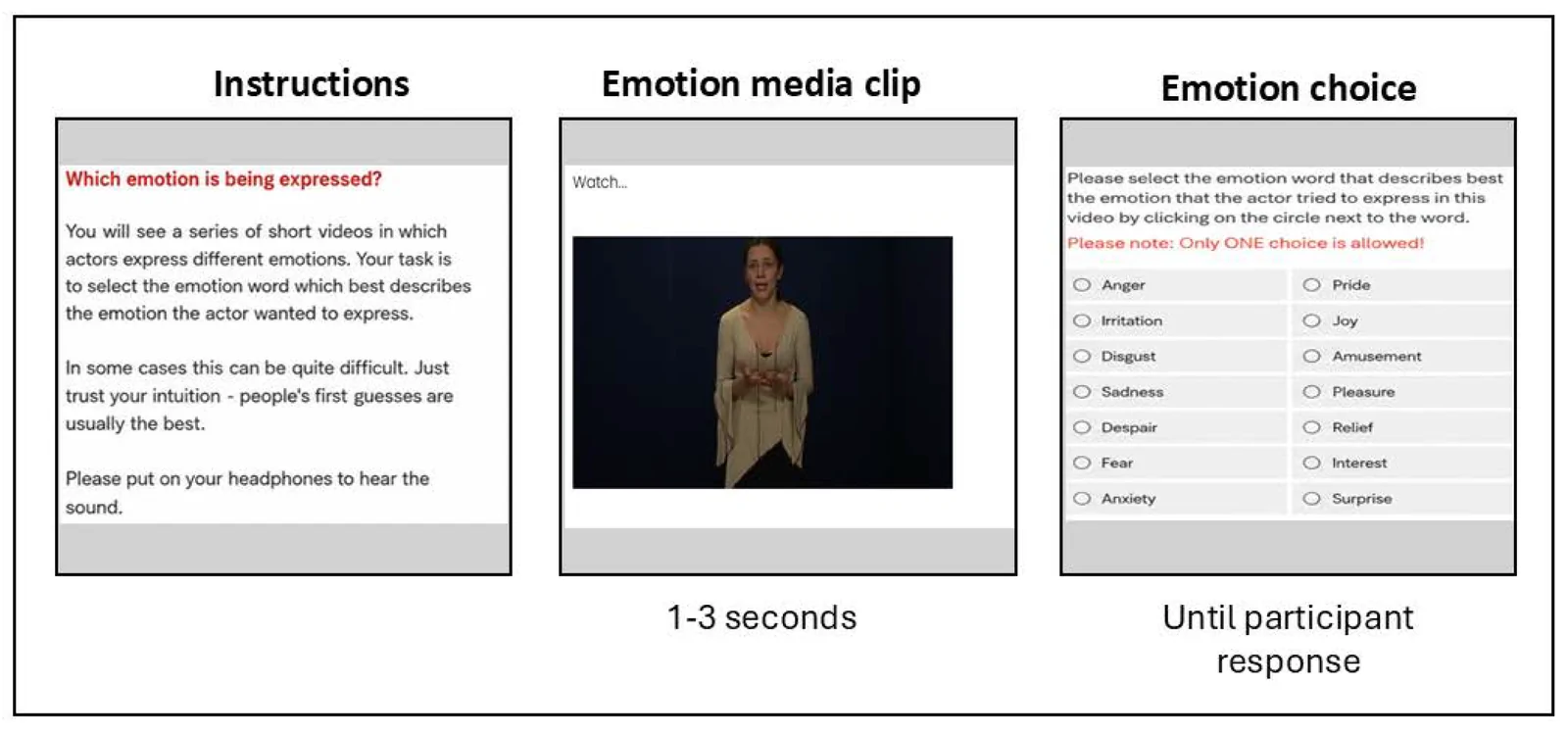
Schematic of the emotion-recognition task structure. Instructions were presented at the start of the task, after which participants viewed blocks of emotion media clips in one of three modalities: visual, auditory, or audiovisual. Following each clip (1–3 s), participants selected the emotion that best matched the actor’s expression from a list of 14 emotion labels. The response screen remained visible until a selection was made. The stimulus set included six positive emotions (amusement, joy, pride, pleasure, relief, interest), seven negative emotions (anger, irritation, disgust, sadness, despair, fear, anxiety), and one neutral emotion (surprise).

The study was administered entirely online using Qualtrics. After reading brief instructions (see Figure 2), participants completed two practice trials to familiarize themselves with the response format. On each trial, they viewed or listened to a short emotional expression that was presented in blocks of either an auditory-only, visual-only, or audiovisual format. Modality blocks were presented in the same pseudo-random sequence for all the participants to avoid a simple repeated modality order. Trial order was randomized within each block. Participants were required to watch or listen to the entire media clip. Once the video ended, participants pressed a button to proceed and were presented with a response screen displaying the 14 emotion labels (see Figure 2), accompanied by the prompt: ‘Please select the emotion word that describes best the emotion that the actor tried to express in this video.’ Only one response was allowed per trial. Participants received course credit upon completing the study.

### 2.4. Analysis

All analyses were conducted using custom scripts in MATLAB (Version R2023a; [15]) and R (Version 4.4.1 [16]). Data and analysis scripts are available on the OSF (https://osf.io/vk4hw/). The primary goal of the analysis was to examine perceived similarity among emotions using representational similarity analysis (RSA), allowing us to investigate how emotions cluster or diverge based on participants’ judgments across auditory, visual, and audiovisual modalities.

#### 2.4.1. Recognition Accuracy

Recognition accuracy was analyzed at the individual-trial level using a binomial generalized linear mixed-effects model (logistic mixed-effects regression), conducted in R with the lme4 package (Version 1.1-37; [17]). The primary model included modality and stimulus version as fixed effects, with crossed random intercepts for participant and stimulus item. A maximal model that additionally included by-participant random slopes for modality yielded a singular fit and was therefore reduced to a random intercepts model. Each stimulus item represented a unique portrayal within a test version. A second model tested whether the effect of modality differed between positive and negative emotions. Surprise was excluded because it was the only emotion of neutral valence. Overall effects were tested with Wald’s chi-square tests, followed by Tukey-adjusted pairwise comparisons. Estimated marginal means were obtained with the emmeans package (Version 1.11.0; [18]) and are reported as population-averaged probabilities.

#### 2.4.2. Error-Only Confusion Profiles and Representational Dissimilarity Matrices

For each modality, 14 × 14 confusion matrices were constructed by pooling valid responses across the two independent stimulus versions. Rows represented target emotions and columns represented participant-selected emotion labels. To reduce the contribution of recognition accuracy to the representational analysis, the primary analysis used error-only confusion profiles. For each target emotion, the correct-response diagonal was removed, and the remaining response counts were normalized to sum to 1, representing the relative pattern of incorrect responses. Pairwise dissimilarities between error-only profiles were computed using correlational distances (1—Pearson’s *r*), yielding a 14 × 14 representational dissimilarity matrix (RDM) for each modality [19]. The full-response profiles, including the correct-response diagonal, were computed in parallel and retained as a sensitivity analysis in the Supplementary Materials (Tables S3–S5).

#### 2.4.3. RDM Reliability and Within-Modality Structure

RDM reliability was estimated using 1000 repeated random split-halves of participants. For each iteration, participants were divided into two groups separately within each stimulus version, an RDM was reconstructed from each half, and reliability was quantified as Spearman’s correlation between the unique off-diagonal elements of the two RDMs. Split-half correlations were corrected using the Spearman–Brown formula [20,21].

Hierarchical clustering was performed directly on each error-only RDM using average linkage [22,23]. The number of clusters was selected by maximizing the mean silhouette value over *k* = 2–10 [24,25]. Cluster stability was assessed using 1000 participant bootstrap samples. For each iteration, participants were resampled with replacement separately within stimulus versions, the RDM was reconstructed, and the clustering analysis was repeated. Stability was quantified using the adjusted Rand index (ARI; [26]) between each bootstrap partition and the full-sample partition, as well as co-assignment probabilities for individual emotion pairs. Silhouette values were interpreted descriptively because solutions containing singleton clusters can limit their usefulness as measures of cluster cohesion.

Two additional measures were used to characterize the error-only RDMs directly. Emotion distinctiveness was assessed within each modality using each emotion’s nearest-neighbor dissimilarity and its mean dissimilarity from all other emotions. Valence organization was assessed by comparing mean dissimilarity between emotions of different valence with mean dissimilarity between emotions of the same valence. Surprise was excluded because it was the only neutral emotion. Statistical significance was evaluated using 5000 random permutations of the valence labels. Positive between-minus-within values indicated greater organization by valence, and permutation *p* values were calculated using the +1 correction.

As a complementary analysis, metric multidimensional scaling (MDS) was applied to the same correlation distance RDM used for hierarchical clustering. MDS projected the emotions into a lower-dimensional space while preserving the pairwise dissimilarities in the original RDM as closely as possible. The primary solution used two dimensions. Euclidean distances were calculated between emotions based on their two-dimensional MDS coordinates, and Ward’s hierarchical clustering was applied to these distances [27]. The number of clusters was again selected from *k* = 2–10 using the silhouette criterion. Agreement between the RDM and MDS-based clustering solutions was quantified using the ARI. Stability of the MDS-based clustering solution was assessed using the same participant-bootstrap procedure described above.

MDS fit was evaluated using metric stress for solutions ranging from one to eight dimensions. To determine whether the clustering structure depended on the dimensionality of the MDS representation, higher-dimensional solutions were reclustered using the same number of clusters as the two-dimensional solution, and agreement with the two-dimensional partition was quantified using the ARI.

#### 2.4.4. Between-Modality Comparisons

Continuous correspondence between modality-specific error-only RDMs was quantified using Spearman’s correlations across their unique off-diagonal elements. Statistical significance was assessed with 10,000 emotion-label permutations of one RDM in each pair. One-sided tests were used because these analyses tested whether cross-modal correspondence was greater than expected under random label alignment. Permutation *p* values used the standard +1 correction. Error-only correlations were corrected for measurement reliability using the corresponding split-half reliability estimates. Agreement between the hierarchical clustering solutions across modalities was assessed using the adjusted Rand index, the Jaccard index for pairs of emotions assigned to the same cluster, and the proportion of each emotion’s same-cluster neighbors retained across modalities.

#### 2.4.5. Leave-One-Modality-Out Comparison

To directly test the relative correspondence of each modality with structure shared by the other modalities, a leave-one-modality-out (LOMO) analysis was performed on the error-only RDMs. For each target modality, the reference RDM was the element-wise mean of the other two modality RDMs. The target RDM was correlated with this reference using Spearman’s rho over the unique off-diagonal elements. Uncertainty was estimated with 5000 paired-participant bootstrap samples. Participants were resampled with replacement separately within stimulus version and carried all three modality responses together, preserving the paired multimodal structure.

Direct modality differences (audiovisual–visual, visual–auditory, and audiovisual–auditory) were tested with 10,000 paired modality-label permutations. Within each permutation and contrast, the two relevant modality-specific participant-count matrices were randomly retained or swapped within participants, after which the error-only RDMs and LOMO references were rebuilt. Tests were two-sided; Monte Carlo *p* values used the +1 convention; and the three pairwise comparisons were adjusted using the Holm procedure. As a separate stability analysis, the LOMO procedure was repeated over 1000 stratified 80/20 participant splits. For each split, the reference was estimated from the two non-target modalities in the training sample and correlated with the target-modality RDM from the held-out sample. These repeated holdout results were summarized descriptively rather than treated as bootstrap inference.

## 3. Results

**Figure 3:**
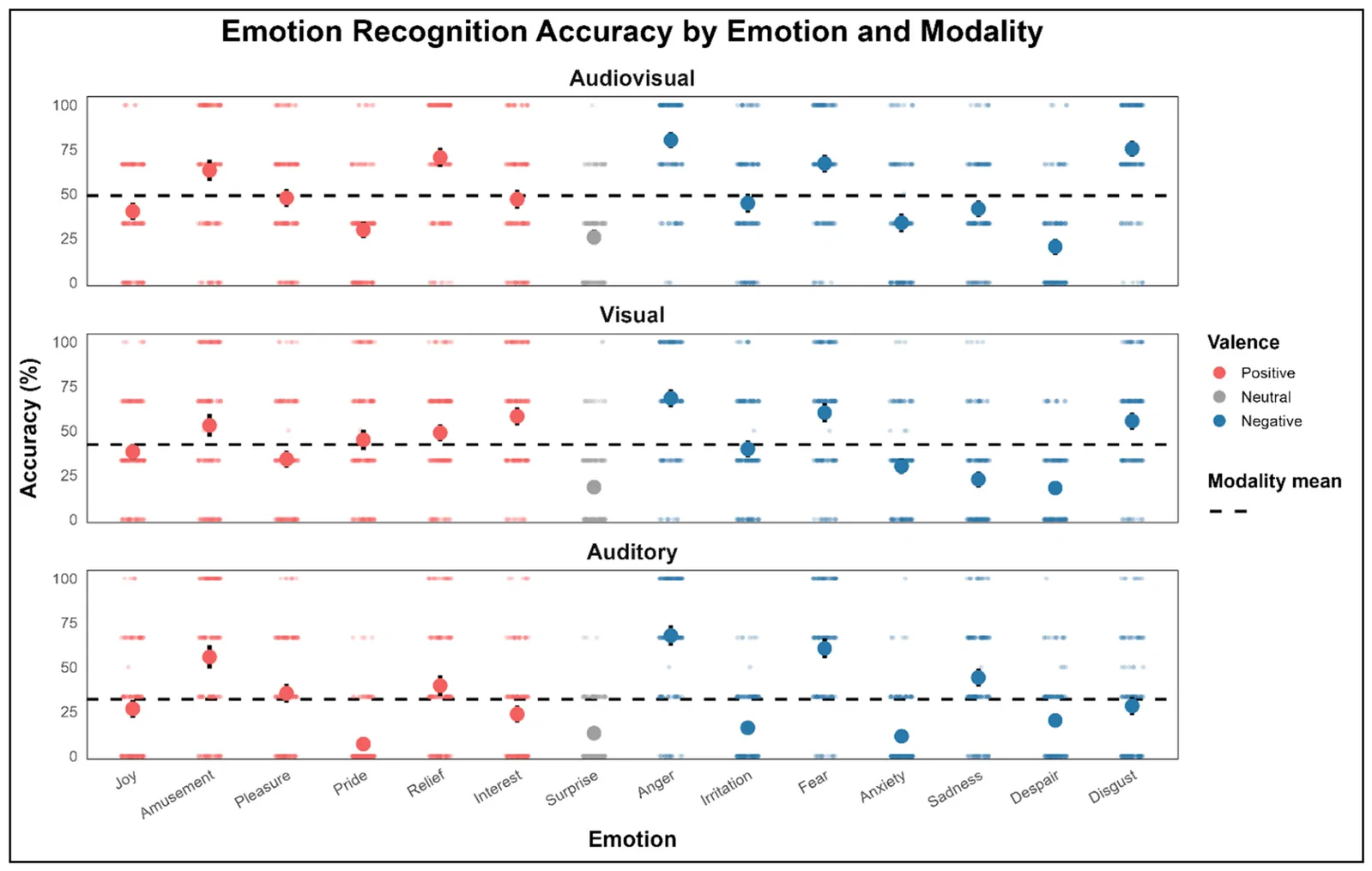
Emotion recognition accuracy by emotion and modality. Mean accuracy scores (colored circles) with 95% confidence intervals (black vertical lines) are shown for each emotion across audiovisual, visual, and auditory conditions. Individual participant accuracies are plotted as semi-transparent points, which fall into discrete bands (0%, 33%, 67%, 100%) because they represent the proportion of correct trials within each emotion–modality combination. Dashed horizontal lines indicate the overall mean accuracy for each modality. Emotions are color-coded by valence: positive (red), neutral (gray), and negative (blue).

Recognition accuracy differed significantly across modalities, χ^2^(2) = 26.58, *p* < 0.001. Accuracy was 49.1% for audiovisual stimuli, 42.2% for visual stimuli, and 31.9% for auditory stimuli (Figure 3). Tukey-adjusted pairwise comparisons showed higher odds of a correct response for audiovisual than auditory trials (OR = 2.89, 95% CI [1.77, 4.72], *p* < 0.001) and for visual than auditory trials (OR = 2.04, 95% CI [1.25, 3.33], *p* = 0.002). Audiovisual and visual accuracy did not differ significantly (OR = 1.42, 95% CI [0.87, 2.31], *p* = 0.215). Accuracy did not differ between the two stimulus versions, χ^2^(1) = 0.18, *p* = 0.669.

The secondary model that tested whether the effect of modality differed between positive and negative emotions reproduced the modality effect, χ^2^(2) = 27.16, *p* < 0.001, but showed no main effect of valence, χ^2^(1) = 0.01, *p* = 0.907, and no modality x valence interaction, χ^2^(2) = 1.06, *p* = 0.589. This indicates that the effect of modality on recognition accuracy was similar for positive and negative emotions. Detailed accuracy statistics are provided in the Supplementary Materials, Table S2.

### 3.1. Reliability of the Representational Structure

The error-only RDMs for each modality are presented in the Supplementary Materials, Tables S7–S9. Split-half reliability was high, with Spearman–Brown-corrected estimates of 0.87 for audiovisual, 0.91 for visual, and 0.86 for auditory RDMs. Reliability was higher for the corresponding full-response RDMs (Supplementary Materials, Table S6), as expected when the correct-response diagonal was retained. The error-only and full-response cluster partitions showed little agreement. RSA ARIs were 0.15, −0.05, and 0.15 for audiovisual, visual, and auditory modalities, respectively, and MDS ARIs were −0.06, −0.05, and 0.22 (Supplementary Materials, Table S10). These values indicate little to no correspondence between the two sets of partitions. Thus, removing the correct-response diagonal substantially altered the discrete clustering solutions, despite the high split-half reliability of the error-only RDMs.

### 3.2. Within-Modality Clustering and MDS Validation

Average-linkage clustering of the error-only RDMs selected *k* = 10 for all three modalities based on mean silhouette values across candidate solutions from *k* = 2–10 (Supplementary Materials, Table S11). With *k* = 10, mean silhouette values were 0.76 for audiovisual, 0.80 for visual, and 0.82 for auditory (Supplementary Materials, Table S12). Since the solutions contained a large number of singleton clusters, these values were interpreted descriptively rather than as evidence of strong cluster cohesion.

The RDM-based clustering solutions were generally stable under participant resampling. Mean bootstrap ARIs relative to the full sample partition were 0.81, 95% CI [0.37, 1.00], for audiovisual; 0.82, 95% CI [0.42, 1.00], for visual; and 0.91, 95% CI [0.58, 1.00], for auditory (Supplementary Materials, Table S13). Thus, the broad partitioning of the error-based representational structure was reproducible across resampled participant sets, although the width of the confidence intervals indicated variability across individual bootstrap samples.

As a complementary analysis, clustering of the two-dimensional MDS configurations also selected k = 10 in each modality, with mean silhouette values of 0.72, 0.70, and 0.74 for audiovisual, visual, and auditory, respectively. Agreement between the RDM-based and MDS-based partitions was ARI = 0.79 for audiovisual and ARI = 0.65 for both visual and auditory modalities. Bootstrap stability of the MDS-based partitions was lower than that of the direct RDM-based solutions, with mean ARIs of 0.58, 95% CI [0.18, 1.00], for audiovisual; 0.67, 95% CI [0.22, 1.00], for visual; and 0.61, 95% CI [0.18, 1.00], for auditory.

**Figure 4:**
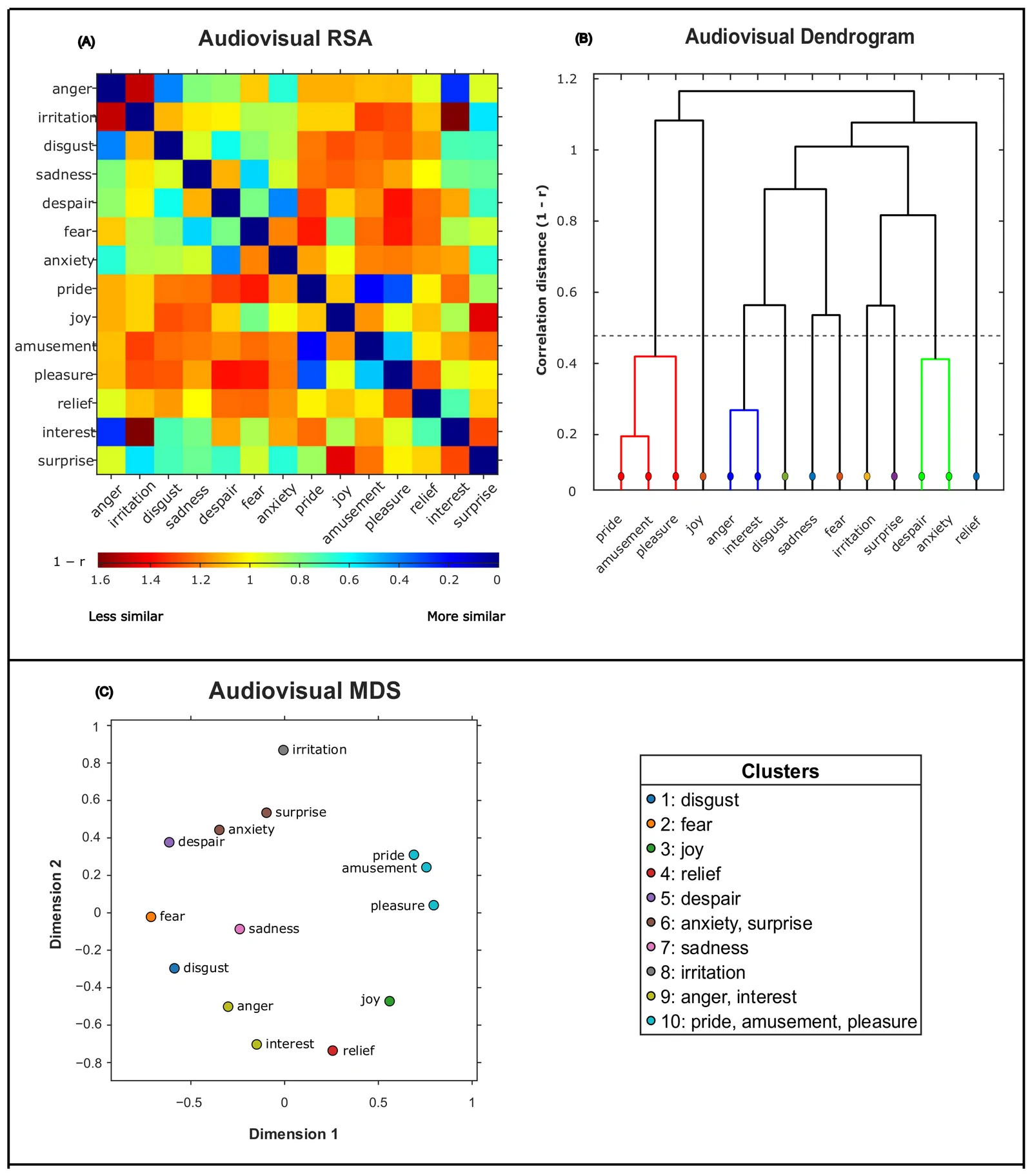
Audiovisual error-only representational structure. Panel (**A**) shows the error-only representational dissimilarity matrix (RDM), computed from response distributions conditional on an incorrect response. Panel (**B**) shows average-linkage hierarchical clustering of the RDM; the number of clusters was selected by maximizing mean silhouette over k = 2–10. The dashed horizontal line indicates the height at which the dendrogram was cut to yield the selected clusters, and branch colors denote cluster membership. Panel (**C**) shows the two-dimensional metric MDS configuration of the same RDM with Ward’s clustering of the MDS coordinates. Point colors indicate cluster membership as in Panel (**B**). The MDS display is used as a visualization and corroborating partition rather than as a complete low-dimensional representation of the RDM.

**Figure 5:**
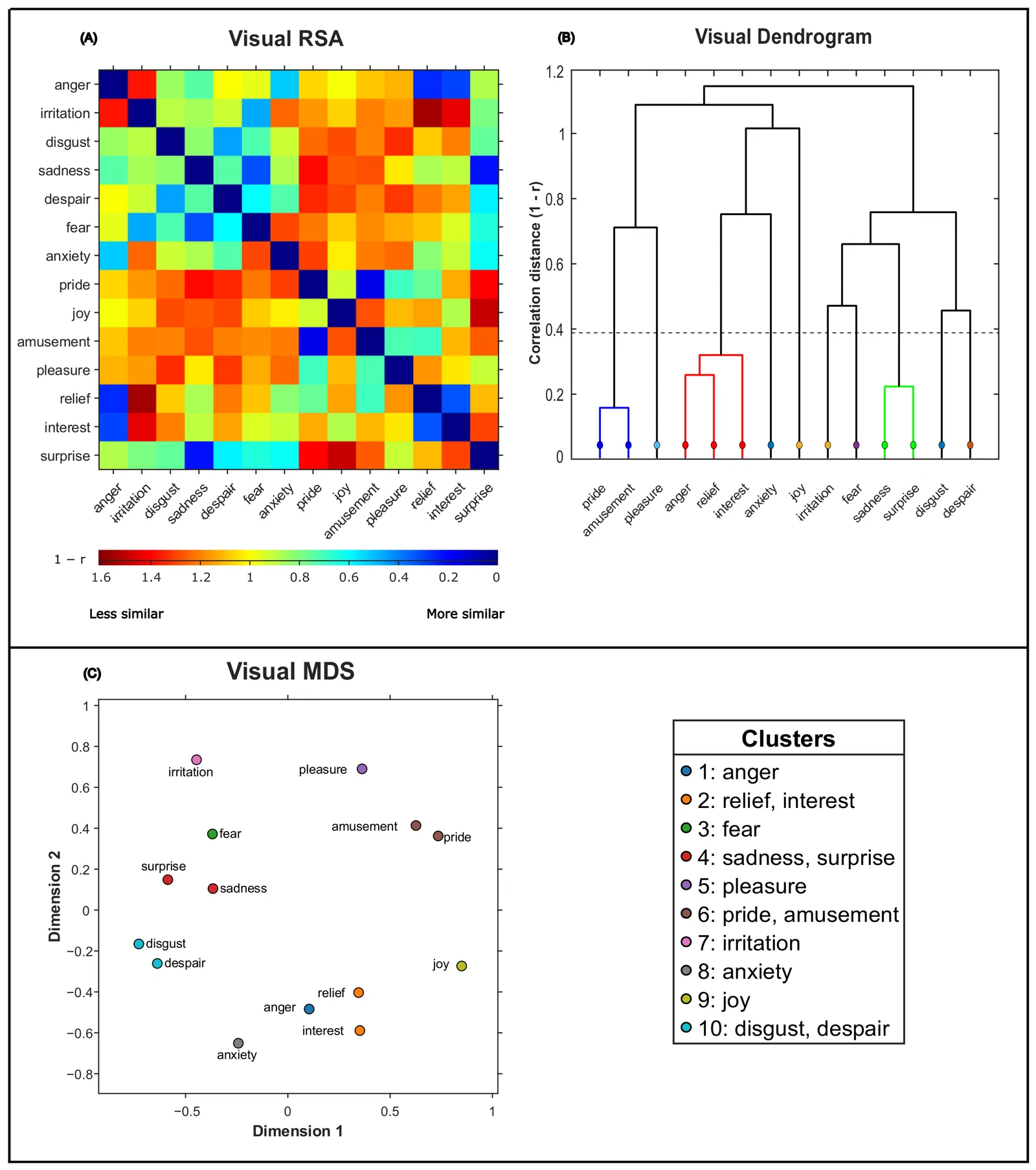
Visual error-only representational structure. Panel (**A**) shows the error-only representational dissimilarity matrix (RDM), computed from response distributions conditional on an incorrect response. Panel (**B**) shows average-linkage hierarchical clustering of the RDM. The number of clusters was selected by maximizing mean silhouette over k = 2–10. The dashed horizontal line indicates the height at which the dendrogram was cut to yield the selected clusters, and branch colors denote cluster membership. Panel (**C**) shows the two-dimensional metric MDS configuration of the same RDM with Ward’s clustering of the MDS coordinates. Point colors indicate cluster membership as in Panel (**B**). The MDS display is used as a visualization and corroborating partition rather than as a complete low-dimensional representation of the RDM.

**Figure 6:**
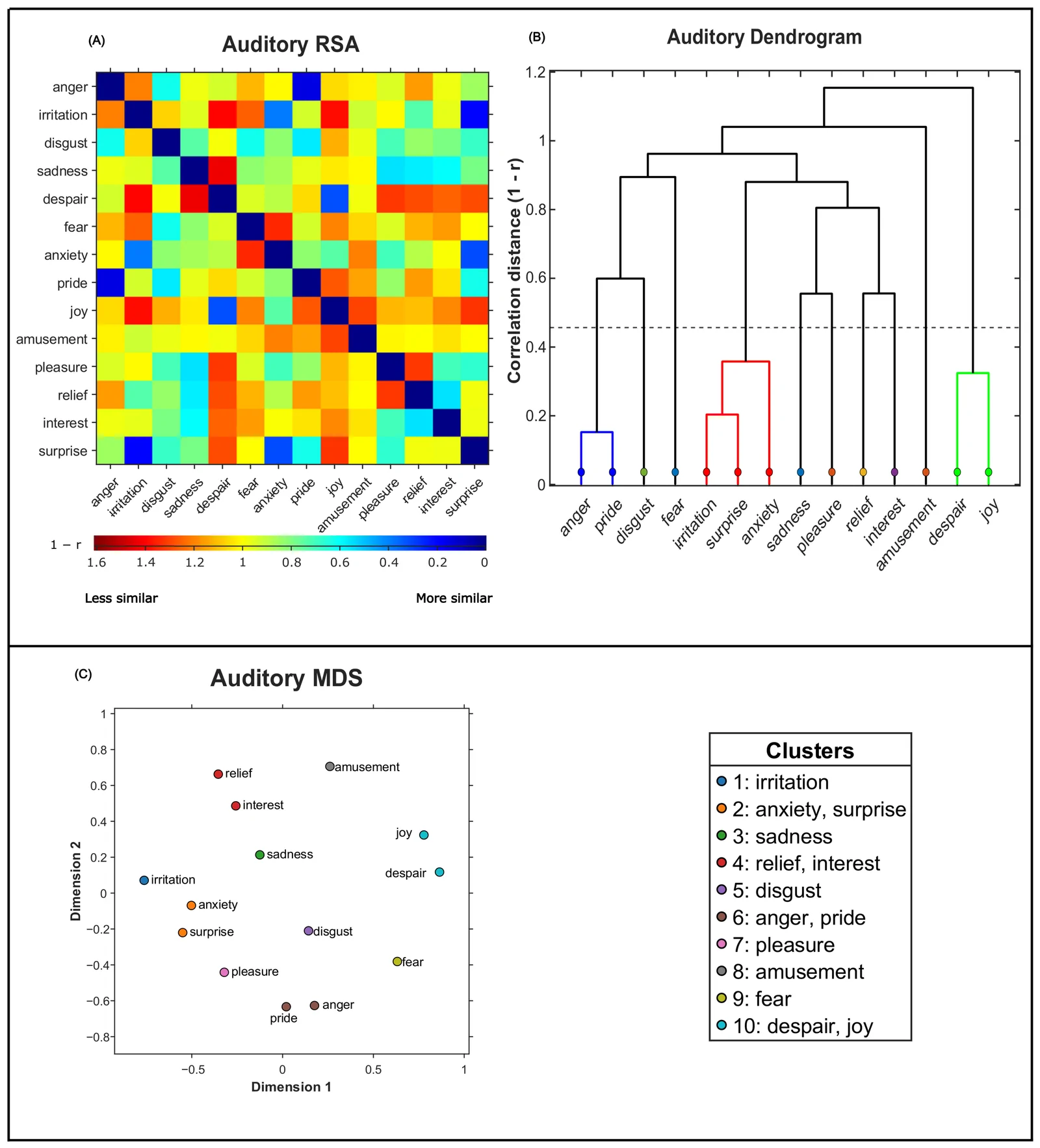
Auditory error-only representational structure. Panel (**A**) shows the error-only representational dissimilarity matrix (RDM), computed from response distributions conditional on an incorrect response. Panel (**B**) shows average-linkage hierarchical clustering of the RDM. The number of clusters was selected by maximizing mean silhouette over k = 2–10. The dashed horizontal line indicates the height at which the dendrogram was cut to yield the selected clusters, and branch colors denote cluster membership. Panel (**C**) shows the two-dimensional metric MDS configuration of the same RDM with Ward’s clustering of the MDS coordinates. Point colors indicate cluster membership as in Panel (**B**). The MDS display is used as a visualization and corroborating partition rather than as a complete low-dimensional representation of the RDM.

The two-dimensional MDS solutions had stress values of 0.25 for audiovisual, 0.20 for visual, and 0.26 for auditory, which decreased to 0.15, 0.12, and 0.15, respectively, in three dimensions. Across three- to eight-dimensional solutions, agreement with the corresponding two-dimensional partition ranged from ARI = 0.58–0.79 for audiovisual, 0.65–0.74 for visual, and 0.48–0.65 for auditory (Supplementary Materials, Table S14). These results indicate that the two-dimensional configurations provided a useful visualization of the representational structure but did not fully capture the higher-dimensional RDM geometry. Accordingly, the clustering performed directly on the RDMs was treated as the primary analysis, whereas the MDS solutions were used as complementary representations. Figure 4, Figure 5 and Figure 6 display the representational dissimilarity matrix heatmaps, dendrograms, and MDS configurations for the audiovisual, visual, and auditory modalities, respectively.

### 3.3. Quantitative Organization of the Error-Only Emotion Space

Valence organized the error-only RDMs in the two modalities containing visual information. Mean between-valence dissimilarity exceeded mean within-valence dissimilarity in the audiovisual modality (difference = 0.24, permutation *p* = 0.002) and the visual modality (difference = 0.28, *p* = 0.003), but not in the auditory modality (difference = −0.08, *p* = 0.921). Detailed statistics are reported in the Supplementary Materials, Table S15. The cross-modal nearest-neighbor analysis identified disgust as the most consistently isolated emotion (minimum nearest-neighbor dissimilarity across modalities = 0.41), followed by fear (0.33), pleasure (0.33), despair (0.32), and joy (0.32) (Supplementary Materials, Table S16).

### 3.4. Between-Modality Comparisons of Representational Structure

#### 3.4.1. Pairwise Correspondence Between Error-Only RDMs

**Figure 7:**
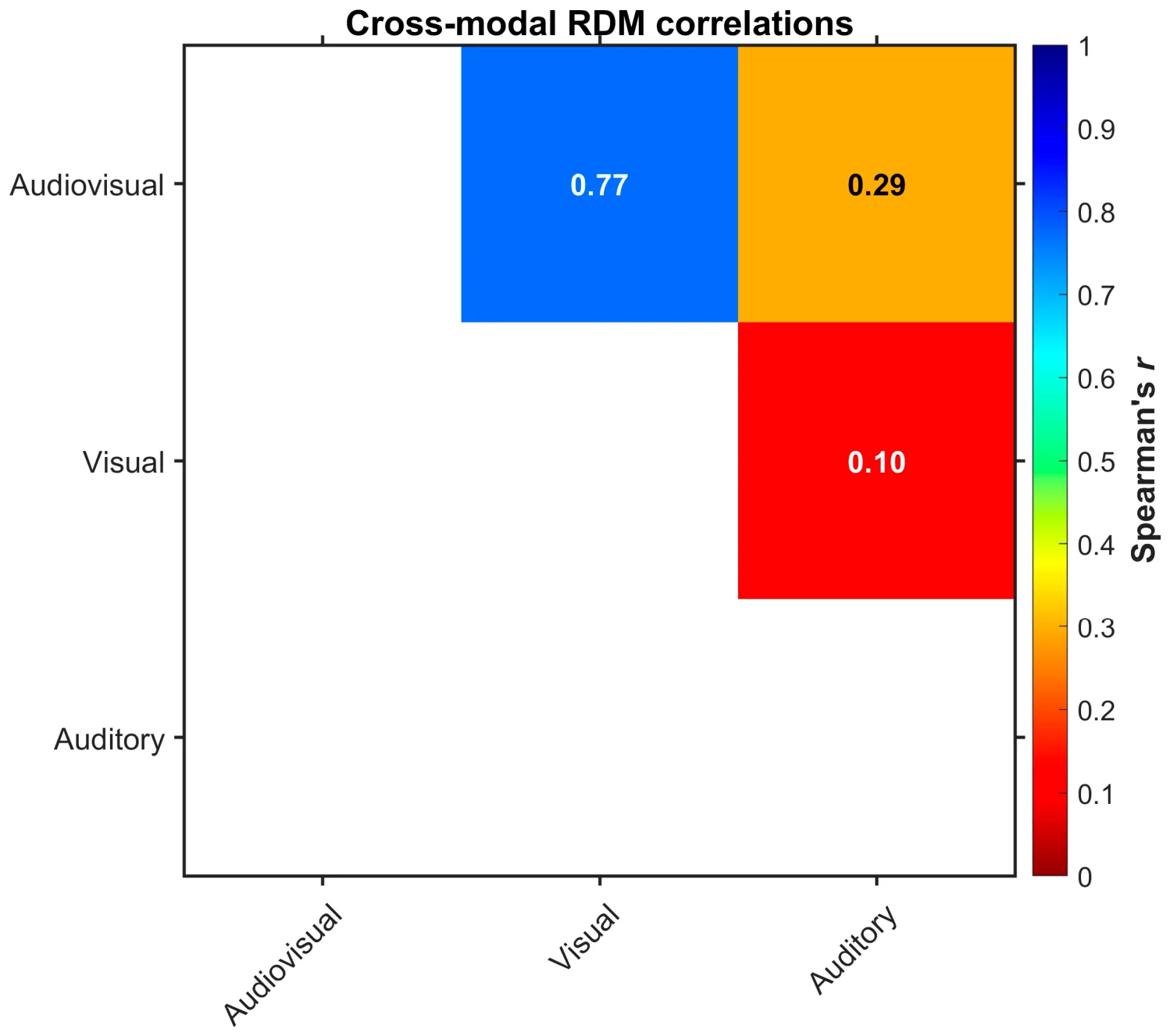
Spearman’s rank correlations among the modality-specific error-only RDMs. Each cell shows Spearman’s correlation between the unique off-diagonal elements of two RDMs. Audiovisual–visual correspondence was strongest (*r*_s_ = 0.77, *p* < 0.001), audiovisual–auditory correspondence was weaker (*r*_s_ = 0.29, *p* = 0.005), and visual–auditory correspondence was not significant (*r*_s_ = 0.10, *p* = 0.174). Permutation *p* values were based on 10,000 emotion-label permutations using the +1 convention.

Pairwise comparisons of the error-only RDMs showed the strongest correspondence between audiovisual and visual modalities (Spearman’s rho (*r*_s_) = 0.77, permutation *p* < 0.001; disattenuated *r*_s_ = 0.87). Audiovisual and auditory RDMs were more weakly but significantly related (*r*_s_ = 0.29, *p* = 0.005; disattenuated *r*_s_ = 0.34), whereas visual and auditory RDMs were not reliably correlated (*r*_s_ = 0.10, *p* = 0.174; disattenuated *r*_s_ = 0.11). The pairwise correspondences among the modality-specific error-only RDMs are shown in Figure 7. For comparison, the full-response RDM correlations were 0.89 for audiovisual–visual, 0.31 for audiovisual–auditory, and 0.26 for visual–auditory, indicating that removal of the correct-response diagonal particularly attenuated the visual–auditory correspondence.

#### 3.4.2. Leave-One-Modality-Out Comparison

The LOMO analysis directly tested how closely each modality corresponded to a reference constructed from the other two modalities. Observed correlations were *r*_s_ = 0.74 for audiovisual, 95% bootstrap CI [0.60, 0.76]; *r*_s_ = 0.55 for visual, 95% CI [0.41, 0.57]; and *r*_s_ = 0.19 for auditory, 95% CI [0.09, 0.26]. All three pairwise differences excluded zero. The audiovisual correspondence exceeded the visual by 0.19, 95% CI [0.10, 0.28]; the visual exceeded the auditory by 0.35, 95% CI [0.21, 0.42]; and the audiovisual exceeded the auditory by 0.55, 95% CI [0.42, 0.59]. Paired modality-label permutation tests confirmed each difference after Holm correction (all adjusted *p* < 0.001), directly supporting the ordering audiovisual > visual > auditory in correspondence with the leave-one-modality-out reference.

The repeated 80/20 holdout analysis showed the same ordering and was used only as a stability check. Across 1000 splits, mean held-out correlations were *M* = 0.59 (*SD* = 0.06) for audiovisual, *M* = 0.45 (*SD* = 0.06) for visual, and *M* = 0.14 (*SD* = 0.05) for auditory.

#### 3.4.3. Cluster-Level Agreement Across Modalities

**Figure 8:**
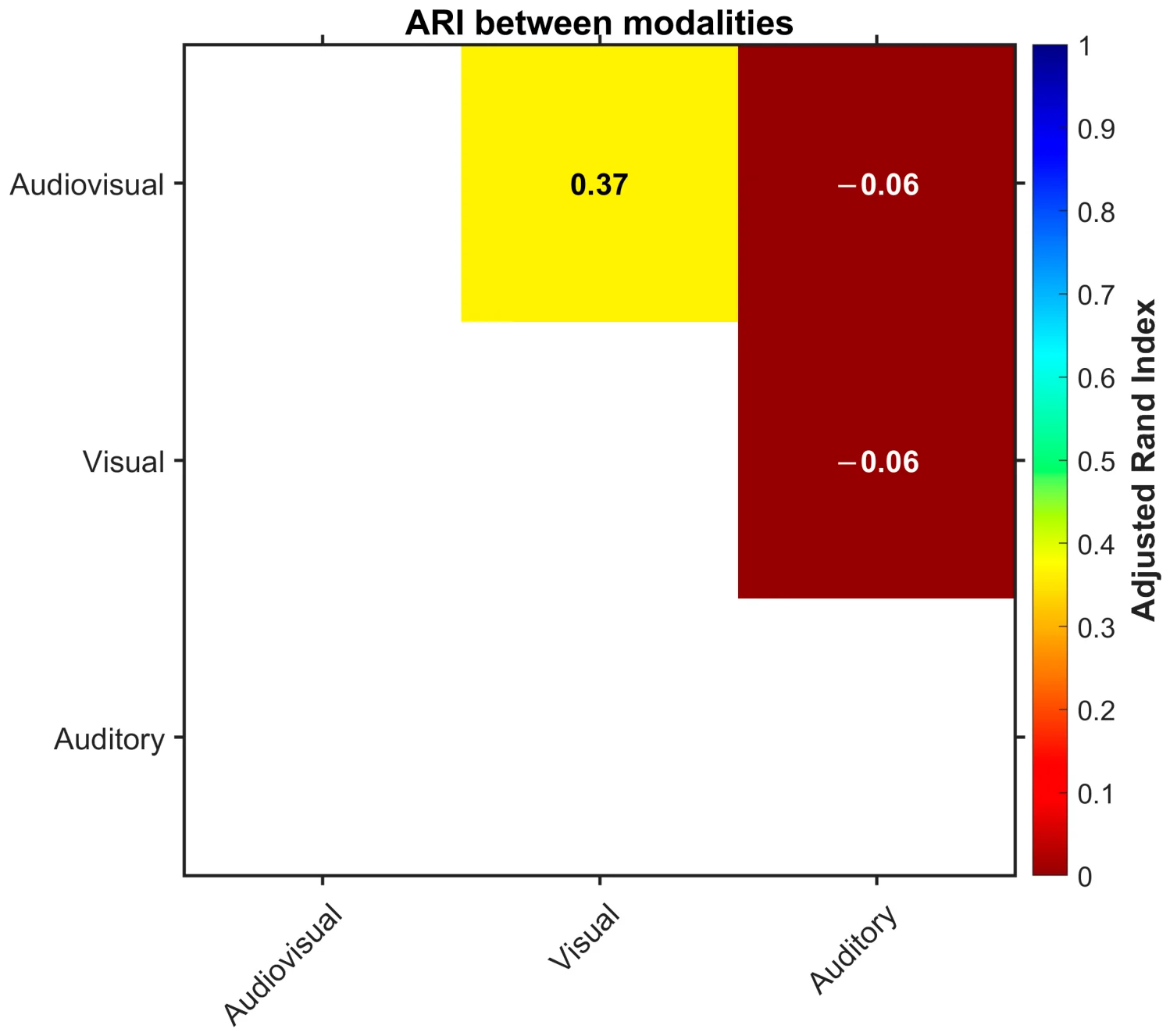
Adjusted Rand index (ARI) between modality-specific hierarchical cluster solutions derived directly from the error-only RDMs. The audiovisual and visual partitions showed modest agreement (ARI = 0.37), whereas audiovisual–auditory and visual–auditory agreement was near chance (both ARI = −0.06). ARI corrects for agreement expected by chance; values near 0 indicate chance-level correspondence and values approaching 1 indicate increasingly similar partitions.

Categorical agreement across the primary RDM-based cluster solutions was modest between audiovisual and visual modalities (ARI = 0.37; Jaccard co-assignment = 0.25) and was no greater than chance for comparisons involving auditory structure (audiovisual–auditory ARI = −0.06, Jaccard = 0; visual–auditory ARI = −0.06, Jaccard = 0). The adjusted Rand indices between the modality-specific cluster solutions are shown in Figure 8. At the level of individual emotions, no non-singleton emotion retained more than one sixth of its same-cluster neighbors across modality pairs. Disgust and fear were singletons in all three modalities (Supplementary Materials, Table S17). These results provided little evidence for a modality-invariant discrete cluster core.

## 4. Discussion

Emotions are often categorized along dimensions, such as by valence and arousal [5,6] or by facial or vocal features [1,2,3,4]. While these dimensions are well-established, they are typically derived from studies using single-modality presentations. It remains unclear whether these emotion categories remain stable across different modalities of presentation. The present study compared recognition accuracy and the representational structure of emotion confusions across auditory, visual, and audiovisual modalities. Recognition accuracy was lower for auditory presentation than for visual and audiovisual presentations, which did not differ from one another. Representational analyses similarly revealed greater correspondence between the visual and audiovisual modalities, whereas auditory emotion confusions showed a particularly distinct organization. Valence structured the audiovisual and visual representational spaces but not the auditory space. In the leave-one-modality-out analysis, audiovisual representations showed the strongest correspondence with a reference derived from the other two modalities, followed by visual and then auditory representations. Together, these findings suggest that representational structure varies across sensory modalities, with greater similarity between the two modalities containing visual information than between either of these modalities and the auditory modality.

Recognition accuracy was significantly lower in the auditory modality than in the visual and audiovisual modalities, which did not differ significantly from one another. Auditory emotion recognition depends on a combination of acoustic cues—including pitch, intensity, and spectral characteristics—that unfold over time and vary in the diagnostic information they provide across emotion categories [9,11,28]. Facial expressions, in contrast, provide spatially distributed visual cues that can directly differentiate emotion categories [29,30], which may make recognition less difficult when visual information is available. The absence of a significant difference between the visual and audiovisual conditions provides no evidence that the addition of auditory information conferred an accuracy advantage over visual information alone in the present task. Taken together, the accuracy results indicate that auditory emotion recognition was more difficult than recognition when visual information was available, and that this pattern was similar across positive and negative emotions.

The audiovisual and visual modalities likewise showed greater similarity in their representational structure. The audiovisual and visual error-only RDMs were strongly correlated. In contrast, the auditory RDM showed only weak correspondence with the audiovisual RDM and no reliable correspondence with the visual RDM. This broader similarity between the audiovisual and visual modalities was also reflected in their valence organization. Between-valence dissimilarity was greater than within-valence dissimilarity in both modalities containing visual information, whereas no such organization was evident in the auditory modality. Cluster-level comparisons showed a similar pattern, with some agreement between audiovisual and visual partitions but little agreement in comparisons involving the auditory modality. Importantly, these differences were observed in the context of reliable within-modality representational structure. The error-only RDMs showed good split-half reliability, and hierarchical clustering and multidimensional scaling produced broadly convergent partitions, although the two-dimensional MDS solutions did not fully capture the structure of the RDMs.

The leave-one-modality-out analysis provided a complementary test of cross-modal correspondence. For each modality, its representational structure was compared with a reference constructed from the other two modalities. The audiovisual structure showed the strongest correspondence with its reference, followed by the visual, and then the auditory structure, with reliable differences between all three modalities. The relatively strong correspondence of the audiovisual modality is consistent with the close relationship observed between the audiovisual and visual RDMs. Together with the absence of a significant accuracy difference between these conditions, this pattern suggests that visual information may contribute substantially to the organization of audiovisual emotion representations, consistent with evidence that facial cues can exert a stronger influence than vocal cues during audiovisual emotion recognition [14]. However, these findings do not establish that audiovisual representations are primarily visual or that vocal information makes no contribution. Rather, they indicate that the audiovisual structure more closely resembled the visual than the auditory structure.

The auditory modality showed a contrasting pattern, with the weakest correspondence to its leave-one-modality-out reference and, unlike the two modalities containing visual information, no evidence of valence organization. One possible explanation is that auditory emotion confusions are structured more strongly by acoustic properties associated with arousal than by valence. Prosodic information appears to contribute particularly strongly to judgments of emotional arousal [31], and acoustic analyses of vocal emotion have shown that arousal accounts for substantial variation in vocal parameters, although valence-related information is also present [32]. Several features of the auditory confusion patterns were descriptively consistent with this possibility. For example, pride was frequently confused with irritation and anger, and joy was frequently confused with fear when emotions were presented auditorily. These errors crossed valence boundaries while occurring between emotions that can share similar levels of arousal. More broadly, the absence of valence organization in the auditory RDM suggests that valence played a weaker organizing role in auditory confusions than in the visual and audiovisual modalities. An emotion space structured more strongly by arousal-related acoustic information could, therefore, contribute to the weaker correspondence between auditory and visual containing representations observed here. However, because arousal was not measured directly and the present analyses characterize forced-choice confusion patterns rather than their underlying perceptual dimensions, this account remains a hypothesis for future investigation.

The present study also illustrates the value of separating which emotions are confused from how often errors occur. Since recognition accuracy is embedded in full confusion profiles, the primary representational analyses were based on error-only profiles, allowing the structure of incorrect responses to be examined while reducing the contribution of overall recognition accuracy. The resulting RDMs showed good split-half reliability, and full-response profiles were retained as a sensitivity analysis to assess the influence of the correct-response diagonal. The robustness of the findings was further evaluated using several complementary quantitative approaches, including permutation tests of cross-modal correspondence; resampling-based assessments of clustering stability; paired bootstrap and permutation procedures for the leave-one-modality-out comparisons; and direct measures of emotion distinctiveness. Together, these analyses show that modality differences extend beyond recognition accuracy to the organization of the errors themselves. The representational structure of audiovisual and visual emotion confusions showed substantial correspondence, whereas auditory confusions were organized in a partly different manner.

These findings should be interpreted within the constraints of the present design, sample, and stimulus set. The task used a closed-set forced-choice format with fourteen emotion labels, such that the resulting dissimilarities reflect patterns of response confusion rather than direct measures of perceptual or neural similarity. The mechanistic interpretations offered above should, therefore, be considered provisional. In addition, each emotion was represented by only three portrayals per modality within a stimulus version, limiting the extent to which the resulting structure can be separated from the portrayals sampled. Stimuli were drawn from a finite actor pool, actor identity was not systematically matched across modalities, and stimulus selection involved subjective judgment. Consequently, modality differences may partly reflect characteristics of the selected portrayals rather than modality alone. Since the study was conducted online, variation in participants’ audio equipment, display characteristics, and listening or viewing environments could also have introduced uncontrolled variability. The sample consisted primarily of young female undergraduate participants judging brief, acted pseudo-utterances and emotional expressions. Generalization should, therefore, be restricted to the present stimulus and label set and may not extend to other populations, spontaneous expressions, or more naturalistic emotional communication. The representational analyses also characterize group-level rather than individual-level organization, as the RDMs were estimated from responses aggregated across participants. In addition, clustering provides a discrete summary of an underlying continuous structure and can vary with analytic choices. However, the primary clustering solutions showed reasonable resampling stability and were broadly supported by the complementary MDS analyses. Future work could examine individual differences in representational structure and directly test whether acoustic features associated with arousal account for the auditory organization observed here.

## 5. Conclusions

This study highlights the role of modality in shaping recognition and representational structure of emotion. Recognition accuracy was lower for auditory presentation than for visual and audiovisual presentations, which did not differ from each other. The structure of emotion confusions showed a parallel pattern where the audiovisual and visual modalities shared a largely valence-based organization, while the auditory modality diverged from both and showed no valence organization. These findings show that how emotions are grouped and confused depends on the sensory modality through which they are perceived. By analyzing the error-only confusion structure and comparing each modality with a reference derived from the other two, the present study showed that auditory emotion representations were less closely aligned with the audiovisual and visual structures, which showed greater correspondence with one another. This divergence may reflect a stronger organization by arousal than by valence. Taken together, these results caution against generalizing emotion recognition outcomes across modalities and underscore the value of moving beyond accuracy to the representational structure of emotion perception.

## Data Availability

The data supporting the findings of this study are available on the Open Science Framework. OSF link: https://osf.io/vk4hw/ (accessed on 28 August 2026).

## Supplementary Materials

The following supporting information can be downloaded at: https://www.mdpi.com/article/10.3390/brainsci16090935/s1. Table S1: Number of scorable responses contributing to each emotion in each modality. Table S2: Recognition accuracy (% correct) for each emotion in each modality. Table S3: Full-response representational dissimilarity matrix for the audiovisual modality. Table S4: Full-response representational dissimilarity matrix for the visual modality. Table S5: Full-response representational dissimilarity matrix for the auditory modality. Table S6: Cross-modal correspondence among the full-response RDMs. Table S7: Error-only representational dissimilarity matrix, audiovisual modality. Table S8: Error-only representational dissimilarity matrix, visual modality. Table S9: Error-only representational dissimilarity matrix, auditory modality. Table S10: Split-half RDM reliability for the error-only and full-response RDMs, and the adjusted Rand index between the error-only and full-response cluster solutions. Table S11: Mean silhouette value for each candidate number of clusters (k = 2–10) in the error-only, average-linkage (RSA) clustering of each modality. Table S12: Selected number of clusters, mean silhouette, and singleton count for the error-only solutions (RSA and MDS), with two-dimensional MDS stress and RSA–MDS partition agreement (ARI). Table S13: Bootstrap cluster stability (1,000 participant resamples). Table S14: MDS fit and dimensionality sensitivity (error-only). Table S15: Valence organization of the error-only representational dissimilarity matrices. Table S16: Emotion distinctiveness within each modality. Table S17: Per-emotion cross-modal neighbour retention across modality pairs.
