# Supplementary material for: Sensory Modality Shapes the Representational Structure of Emotion Recognition
Source: Brain Sci. 2026 Aug 31;16(9):935. doi: 10.3390/brainsci16090935 (PMC13604197; doi:10.3390/brainsci16090935)
Supplement: Supplementary file 1 [file brainsci-16-00935-s001.zip › brainsci-4485860-supplementary.pdf]

Supplemental Materials

***Supplemental to* CHEGE *et al*., 2026**

OSF link to data and analysis scripts: https://osf.io/vk4hw/

"Error-only" denotes RDMs built from off-diagonal (incorrect response) profiles. "Full response" denotes the profiles that include the correct response diagonal.

Sample and stimulus sampling

Table S1

Number of scorable responses contributing to each emotion in each modality

Emotion	Audiovisual	Visual	Auditory
Anger	393	393	393
Irritation	393	392	393
Disgust	393	393	393
Sadness	393	393	393
Despair	393	393	393
Fear	393	393	393
Anxiety	392	393	393
Pride	393	393	393
Joy	392	393	392
Amusement	393	393	393
Pleasure	393	392	393
Relief	393	393	393
Interest	393	393	393
Surprise	392	393	392

Note. N = 131 x approximately 3 exemplars per cell

Recognition accuracy by emotion

Table S2

Recognition accuracy (% correct) for each emotion in each modality

Emotion (valence)	Audiovisual	Visual	Auditory
Anger (Neg)	79.9 [75.7, 83.6]	67.7 [62.9, 72.1]	66.7 [61.9, 71.1]
Irritation (Neg)	44.5 [39.7, 49.5]	39.3 [34.6, 44.2]	16.3 [13.0, 20.3]
Disgust (Neg)	75.3 [70.8, 79.3]	56.7 [51.8, 61.6]	27.2 [23.1, 31.8]
Sadness (Neg)	41.2 [36.5, 46.2]	22.9 [19.0, 27.3]	45.0 [40.2, 50.0]
Despair (Neg)	20.1 [16.4, 24.3]	17.6 [14.1, 21.6]	19.8 [16.2, 24.1]
Fear (Neg)	67.9 [63.2, 72.4]	59.8 [54.9, 64.5]	60.1 [55.1, 64.8]
Anxiety (Neg)	33.4 [28.9, 38.2]	29.5 [25.2, 34.2]	11.7 [8.9, 15.3]
Pride (Pos)	30.3 [25.9, 35.0]	45.8 [40.9, 50.7]	6.9 [4.8, 9.8]

Emotion (valence)	Audiovisual	Visual	Auditory
Joy (Pos)	40.1 [35.3, 45.0]	38.4 [33.7, 43.3]	26.8 [22.6, 31.4]
Amusement (Pos)	62.8 [58.0, 67.5]	53.9 [49.0, 58.8]	54.7 [49.8, 59.6]
Pleasure (Pos)	47.1 [42.2, 52.0]	33.7 [29.2, 38.5]	34.4 [29.8, 39.2]
Relief (Pos)	71.2 [66.6, 75.5]	48.6 [43.7, 53.5]	39.4 [34.7, 44.4]
Interest (Pos)	47.3 [42.4, 52.3]	58.3 [53.3, 63.0]	23.9 [20.0, 28.4]
Surprise (Neu)	25.5 [21.4, 30.0]	19.1 [15.5, 23.3]	13.5 [10.5, 17.3]

Note. CIs are 95% Wilson confidence intervals. Valence is abbreviated Neg/Pos/Neu.

Full response sensitivity analysis

The full response per modality structure is shown in Table S3-S5, and the cross modal correspondence among the full response RDMs reported in Table S6.

Table S3

Full response representational dissimilarity matrix for the audiovisual modality

	ange	irri	disg	sadn	desp	fear	anxi	prid	joy	amus	plea	reli	inte	surp
ange	0.00	0.86	1.07	1.14	1.13	1.07	1.20	1.19	1.18	1.12	1.15	1.11	1.12	1.22
irri	0.86	0.00	0.97	1.01	1.01	1.08	0.92	1.18	1.26	1.21	1.19	1.13	0.77	0.89
disg	1.07	0.97	0.00	1.06	1.22	1.10	1.10	1.21	1.21	1.14	1.19	1.09	1.12	1.08
sadn	1.14	1.01	1.06	0.00	0.69	1.10	1.07	1.27	1.32	1.23	1.22	1.04	1.10	1.07
desp	1.13	1.01	1.22	0.69	0.00	0.33	0.29	1.49	1.27	1.29	1.38	1.24	1.22	0.80
fear	1.07	1.08	1.10	1.10	0.33	0.00	0.48	1.25	1.14	1.15	1.22	1.13	1.16	0.94
anxi	1.20	0.92	1.10	1.07	0.29	0.48	0.00	1.32	1.27	1.25	1.30	1.15	0.94	0.59
prid	1.19	1.18	1.21	1.27	1.49	1.25	1.32	0.00	0.52	0.78	0.91	1.02	0.78	1.23
joy	1.18	1.26	1.21	1.32	1.27	1.14	1.27	0.52	0.00	0.47	0.92	1.13	1.17	0.98
amus	1.12	1.21	1.14	1.23	1.29	1.15	1.25	0.78	0.47	0.00	0.90	1.10	1.10	1.31
plea	1.15	1.19	1.19	1.22	1.38	1.22	1.30	0.91	0.92	0.90	0.00	0.66	0.97	1.27
reli	1.11	1.13	1.09	1.04	1.24	1.13	1.15	1.02	1.13	1.10	0.66	0.00	1.03	1.07
inte	1.12	0.77	1.12	1.10	1.22	1.16	0.94	0.78	1.17	1.10	0.97	1.03	0.00	0.55
surp	1.22	0.89	1.08	1.07	0.80	0.94	0.59	1.23	0.98	1.31	1.27	1.07	0.55	0.00

Note. Values are correlation distances (1 − Spearman/Pearson)

Table S4

Full response representational dissimilarity matrix for the visual modality

	ange	irri	disg	sadn	desp	fear	anxi	prid	joy	amus	plea	reli	inte	surp
ange	0.00	0.56	1.01	1.24	1.20	1.09	1.00	1.10	1.10	1.16	1.24	1.11	1.11	1.19
irri	0.56	0.00	0.93	0.90	1.01	1.08	0.66	1.21	1.13	1.21	1.28	0.93	0.97	0.91
disg	1.01	0.93	0.00	0.98	0.98	1.10	1.08	1.23	1.27	1.19	1.25	1.14	1.08	1.07
sadn	1.24	0.90	0.98	0.00	0.20	1.00	0.61	1.36	1.49	1.32	1.28	1.05	0.86	0.80
desp	1.20	1.01	0.98	0.20	0.00	0.49	0.37	1.40	1.43	1.29	1.43	1.27	1.15	0.79
fear	1.09	1.08	1.10	1.00	0.49	0.00	0.43	1.22	1.13	1.15	1.23	1.17	1.14	0.76
anxi	1.00	0.66	1.08	0.61	0.37	0.43	0.00	1.26	1.31	1.31	1.34	1.15	1.02	0.65

	ange	irri	disg	sadn	desp	fear	anxi	prid	joy	amus	plea	reli	inte	surp
prid	1.10	1.21	1.23	1.36	1.40	1.22	1.26	0.00	0.73	0.80	0.93	1.03	1.06	1.48
joy	1.10	1.13	1.27	1.49	1.43	1.13	1.31	0.73	0.00	0.41	0.92	1.09	1.15	1.16
amus	1.16	1.21	1.19	1.32	1.29	1.15	1.31	0.80	0.41	0.00	0.89	1.07	1.11	1.45
plea	1.24	1.28	1.25	1.28	1.43	1.23	1.34	0.93	0.92	0.89	0.00	0.38	1.02	1.27
reli	1.11	0.93	1.14	1.05	1.27	1.17	1.15	1.03	1.09	1.07	0.38	0.00	1.08	1.02
inte	1.11	0.97	1.08	0.86	1.15	1.14	1.02	1.06	1.15	1.11	1.02	1.08	0.00	0.65
surp	1.19	0.91	1.07	0.80	0.79	0.76	0.65	1.48	1.16	1.45	1.27	1.02	0.65	0.00

Note. Values are correlation distances (1 − Spearman/Pearson)

Table S5

Full response representational dissimilarity matrix for the auditory modality

	ange	irri	disg	sadn	desp	fear	anxi	prid	joy	amus	plea	reli	inte	surp
ange	0.00	1.06	1.11	1.19	1.02	1.06	1.31	0.76	1.16	1.12	1.16	1.19	1.25	1.30
irri	1.06	0.00	1.09	1.13	1.47	1.31	0.60	0.36	1.26	1.10	0.83	0.86	0.26	0.28
disg	1.11	1.09	0.00	0.87	1.16	1.26	1.08	0.95	1.32	1.14	0.98	1.12	1.15	1.30
sadn	1.19	1.13	0.87	0.00	0.64	1.08	0.61	1.09	0.99	1.11	0.79	0.95	0.92	1.00
desp	1.02	1.47	1.16	0.64	0.00	0.38	0.77	1.16	0.59	1.18	1.16	1.34	1.46	1.23
fear	1.06	1.31	1.26	1.08	0.38	0.00	0.71	1.24	0.57	1.15	1.22	1.19	1.25	1.23
anxi	1.31	0.60	1.08	0.61	0.77	0.71	0.00	0.94	1.21	1.31	0.89	0.94	0.44	0.42
prid	0.76	0.36	0.95	1.09	1.16	1.24	0.94	0.00	1.37	1.24	1.10	1.10	0.79	0.72
joy	1.16	1.26	1.32	0.99	0.59	0.57	1.21	1.37	0.00	0.63	1.25	1.25	1.43	1.17
amus	1.12	1.10	1.14	1.11	1.18	1.15	1.31	1.24	0.63	0.00	1.14	1.01	1.17	1.11
plea	1.16	0.83	0.98	0.79	1.16	1.22	0.89	1.10	1.25	1.14	0.00	0.34	0.51	1.06
reli	1.19	0.86	1.12	0.95	1.34	1.19	0.94	1.10	1.25	1.01	0.34	0.00	0.36	0.92
inte	1.25	0.26	1.15	0.92	1.46	1.25	0.44	0.79	1.43	1.17	0.51	0.36	0.00	0.35
surp	1.30	0.28	1.30	1.00	1.23	1.23	0.42	0.72	1.17	1.11	1.06	0.92	0.35	0.00

Note. Values are correlation distances (1 − Spearman/Pearson)

Table S6

Cross-modal correspondence among the full-response RDMs

Modality pair	Spearman r_s	Permutation p	Disattenuated r_s
AV–VIS	0.89	< .001	0.92
AV–AUD	0.31	0.007	0.32
VIS–AUD	0.26	0.018	0.27

Note. Spearman rank correlation over off-diagonal elements; permutation p from 10,000 label permutations; disattenuated for reliability.

Error-only RDMs

Table S7

Error-only RDM, Audiovisual

	ange	irri	disg	sadn	desp	fear	anxi	prid	joy	amus	plea	reli	inte	surp
ange	0.00	1.50	0.41	0.81	0.84	1.09	0.67	1.13	1.13	1.11	1.11	0.95	0.27	0.95
irri	1.50	0.00	1.12	1.05	1.02	0.88	0.88	1.07	1.08	1.31	1.28	1.11	1.61	0.56
disg	0.41	1.12	0.00	0.95	0.63	0.82	0.89	1.22	1.28	1.24	1.27	1.16	0.72	0.71
sadn	0.81	1.05	0.95	0.00	1.12	0.54	0.93	1.23	1.26	1.22	1.15	1.01	0.79	0.77
desp	0.84	1.02	0.63	1.12	0.00	0.80	0.41	1.31	1.08	1.18	1.38	1.24	1.14	0.70
fear	1.09	0.88	0.82	0.54	0.80	0.00	1.20	1.37	0.79	1.24	1.37	1.25	0.88	0.93
anxi	0.67	0.88	0.89	0.93	0.41	1.20	0.00	1.15	0.99	1.20	1.21	1.17	1.15	0.66
prid	1.13	1.07	1.22	1.23	1.31	1.37	1.15	0.00	1.09	0.20	0.33	1.03	1.24	0.85
joy	1.13	1.08	1.28	1.26	1.08	0.79	0.99	1.09	0.00	1.18	0.98	1.11	0.88	1.45
amus	1.11	1.31	1.24	1.22	1.18	1.24	1.20	0.20	1.18	0.00	0.51	1.04	1.15	1.23
plea	1.11	1.28	1.27	1.15	1.38	1.37	1.21	0.33	0.98	0.51	0.00	1.28	0.96	1.02
reli	0.95	1.11	1.16	1.01	1.24	1.25	1.17	1.03	1.11	1.04	1.28	0.00	0.73	1.07
inte	0.27	1.61	0.72	0.79	1.14	0.88	1.15	1.24	0.88	1.15	0.96	0.73	0.00	1.29
surp	0.95	0.56	0.71	0.77	0.70	0.93	0.66	0.85	1.45	1.23	1.02	1.07	1.29	0.00

Note. Values are correlation distances (1 − Spearman/Pearson)

Table S8

Error-only RDM, Visual.

	ange	irri	disg	sadn	desp	fear	anxi	prid	joy	amus	plea	reli	inte	surp
ange	0.00	1.38	0.85	0.74	1.00	0.97	0.52	1.07	0.99	1.09	1.12	0.26	0.31	0.89
irri	1.38	0.00	0.90	0.87	0.93	0.47	1.24	1.17	1.08	1.21	1.16	1.55	1.45	0.80
disg	0.85	0.90	0.00	0.83	0.46	0.73	0.93	1.24	1.29	1.20	1.34	1.08	1.21	0.76
sadn	0.74	0.87	0.83	0.00	0.74	0.33	0.88	1.39	1.26	1.28	1.04	0.87	0.91	0.22
desp	1.00	0.93	0.46	0.74	0.00	0.60	0.74	1.34	1.30	1.21	1.32	1.22	1.15	0.58
fear	0.97	0.47	0.73	0.33	0.60	0.00	1.30	1.22	1.09	1.19	1.14	1.10	0.95	0.65
anxi	0.52	1.24	0.93	0.88	0.74	1.30	0.00	1.31	1.03	1.21	1.24	0.82	0.91	0.62
prid	1.07	1.17	1.24	1.39	1.34	1.22	1.31	0.00	0.93	0.16	0.70	0.77	1.14	1.41
joy	0.99	1.08	1.29	1.26	1.30	1.09	1.03	0.93	0.00	1.28	1.12	1.15	0.89	1.50
amus	1.09	1.21	1.20	1.28	1.21	1.19	1.21	0.16	1.28	0.00	0.72	0.71	1.12	1.26
plea	1.12	1.16	1.34	1.04	1.32	1.14	1.24	0.70	1.12	0.72	0.00	1.18	1.04	0.92
reli	0.26	1.55	1.08	0.87	1.22	1.10	0.82	0.77	1.15	0.71	1.18	0.00	0.33	1.11
inte	0.31	1.45	1.21	0.91	1.15	0.95	0.91	1.14	0.89	1.12	1.04	0.33	0.00	1.30
surp	0.89	0.80	0.76	0.22	0.58	0.65	0.62	1.41	1.50	1.26	0.92	1.11	1.30	0.00

Note. Values are correlation distances (1 − Spearman/Pearson)

Table S9

Error-only RDM, Auditory.

	ange	irri	disg	sadn	desp	fear	anxi	prid	joy	amus	plea	reli	inte	surp
ange	0.00	1.21	0.63	0.98	0.94	1.12	1.01	0.15	1.06	1.02	0.95	1.16	0.98	0.85
irri	1.21	0.00	1.08	0.96	1.40	1.26	0.40	0.94	1.39	0.92	1.01	0.73	0.97	0.20
disg	0.63	1.08	0.00	0.74	0.99	0.63	0.83	0.57	1.13	0.97	0.71	0.84	0.80	0.69
sadn	0.98	0.96	0.74	0.00	1.43	0.83	0.86	0.97	1.04	1.01	0.56	0.58	0.61	0.79

	ange	irri	disg	sadn	desp	fear	anxi	prid	joy	amus	plea	reli	inte	surp
desp	0.94	1.40	0.99	1.43	0.00	0.95	0.90	1.07	0.32	0.98	1.32	1.29	1.25	1.29
fear	1.12	1.26	0.63	0.83	0.95	0.00	1.35	0.94	1.20	1.07	0.95	1.13	1.16	1.01
anxi	1.01	0.40	0.83	0.86	0.90	1.35	0.00	0.83	0.74	1.21	0.71	0.89	1.00	0.32
prid	0.15	0.94	0.57	0.97	1.07	0.94	0.83	0.00	1.27	1.15	0.84	1.16	1.05	0.64
joy	1.06	1.39	1.13	1.04	0.32	1.20	0.74	1.27	0.00	1.30	1.12	1.11	1.20	1.33
amus	1.02	0.92	0.97	1.01	0.98	1.07	1.21	1.15	1.30	0.00	1.02	1.01	1.05	1.01
plea	0.95	1.01	0.71	0.56	1.32	0.95	0.71	0.84	1.12	1.02	0.00	1.32	0.71	0.67
reli	1.16	0.73	0.84	0.58	1.29	1.13	0.89	1.16	1.11	1.01	1.32	0.00	0.56	0.99
inte	0.98	0.97	0.80	0.61	1.25	1.16	1.00	1.05	1.20	1.05	0.71	0.56	0.00	0.98
surp	0.85	0.20	0.69	0.79	1.29	1.01	0.32	0.64	1.33	1.01	0.67	0.99	0.98	0.00

Note. Values are correlation distances (1 − Spearman/Pearson)

RDM reliability and error-only versus full response stability

Table S10

Split-half RDM reliability for the error-only and full response RDMs, and the adjusted Rand index between the error-only and full-response cluster solutions (RSA and MDS)

Modality	Split-half reliability (error-only)	Split-half reliability (full)	RSA ARI (error vs full)	MDS ARI (error vs full)
Audiovisual	0.87	0.97	0.15	-0.06
Visual	0.91	0.97	-0.05	-0.05
Auditory	0.86	0.94	0.15	0.22

Note. Split-half RDM reliability is Spearman-Brown corrected. High reliability with low error-only vs full response ARI indicates that removing the correct response diagonal reliably changes the discrete solution rather than adding noise.

Clustering and MDS validation

Table S11

Mean silhouette value for each candidate number of clusters (k = 2–10) in the error-only, average-linkage (RSA) clustering of each modality.

k (clusters)	Audiovisual	Visual	Auditory
2	0.26	0.30	0.29
3	0.30	0.38	0.29
4	0.34	0.45	0.33
5	0.41	0.42	0.40
6	0.49	0.50	0.50
7	0.52	0.57	0.52
8	0.57	0.62	0.60
9	0.66	0.70	0.70
**10**	**0.76**	**0.80**	**0.82**

Note. For every modality the mean silhouette increased monotonically across the search range, so the selection criterion chose the largest solution examined (k = 10; shown in bold).

Table S12

Selected number of clusters, mean silhouette, and singleton count for the error-only solutions (RSA and MDS), with the two-dimensional MDS stress and the RSA-MDS partition agreement (ARI).

Modality	RSA k	RSA silhouette	RSA singletons	MDS k	MDS silhouette	MDS 2D stress	RSA–MDS ARI
Audiovisual	10	0.76	7	10	0.72	0.25	0.79
Visual	10	0.80	7	10	0.70	0.20	0.65
Auditory	10	0.82	7	10	0.74	0.26	0.65

Note. Silhouette values are influenced by the large number of singleton clusters. Robustness is therefore assessed through resampling.

Table S13

Bootstrap cluster stability (1,000 participant resamples)

Method	Modality	Within-cluster consensus	Between-cluster consensus	ARI to reference [95% CI]
RSA	Audiovisual	0.82	0.01	0.81 [0.37, 1.00]
MDS	Audiovisual	0.60	0.02	0.58 [0.18, 1.00]
RSA	Visual	0.81	0.01	0.82 [0.42, 1.00]
MDS	Visual	0.73	0.02	0.67 [0.22, 1.00]
RSA	Auditory	0.91	0.00	0.91 [0.58, 1.00]
MDS	Auditory	0.66	0.02	0.61 [0.18, 1.00]

Note. Mean co-assignment consensus for pairs grouped together vs. apart in the reference solution, and the ARI between each bootstrap partition and the reference.

Table S14

MDS fit and dimensionality sensitivity (error-only)

Dim	Stress AV	Stress VIS	Stress AUD	ARI-to-2D AV	ARI-to-2D VIS	ARI-to-2D AUD
1	0.46	0.43	0.46	—	—	—
2	0.25	0.20	0.26	1.00	1.00	1.00
3	0.15	0.12	0.15	0.58	0.65	0.48
4	0.10	0.08	0.11	0.58	0.74	0.48
5	0.07	0.07	0.08	0.79	0.65	0.65
6	0.07	0.06	0.07	0.79	0.65	0.65
7	0.07	0.06	0.07	0.79	0.65	0.65
8	0.07	0.06	0.07	0.79	0.65	0.65

Note. Stress indexes the fit of the metric multidimensional scaling (MDS) configuration at each dimensionality. ARI is the adjusted Rand index between the cluster partition recovered at each dimensionality and the primary two-dimensional partition, which serves as the reference (ARI = 1 by definition). One-dimensional solutions were not clustered (—). Lower stress indicates better fit. ARI values below 1 indicate departures from the two-dimensional solution.

Table S15

Valence Organization of Error-Only Representational Dissimilarity Matrices

Modality	Within-valence dissimilarity	Between-valence dissimilarity	Difference	Permutation p
Audiovisual	0.90	1.14	0.24	0.002
Visual	0.85	1.13	0.28	0.003
Auditory	1.01	0.93	-0.08	0.921

Note. Mean within-valence and between-valence dissimilarities were calculated for positive and negative emotions only. Surprise was excluded because it was the only neutral emotion. Difference values represent between-valence minus within-valence dissimilarity, with positive values indicating greater dissimilarity between emotions of different valence than between emotions of the same valence. *p* values were obtained from 5,000 permutations of the valence labels.

Table S16

Emotion Distinctiveness Within Each Modality

Emotion	NN AV	NN VIS	NN AUD	Mean AV	Mean VIS	Mean AUD	Min NN (across mod.)
Anger	0.27	0.26	0.15	0.92	0.86	0.93	0.15
Irritation	0.56	0.47	0.20	1.11	1.09	0.96	0.20
Disgust	0.41	0.46	0.57	0.95	0.98	0.82	0.41
Sadness	0.54	0.22	0.56	0.99	0.87	0.87	0.22
Despair	0.41	0.46	0.32	0.99	0.97	1.09	0.32
Fear	0.54	0.33	0.63	1.01	0.90	1.04	0.33
Anxiety	0.41	0.52	0.32	0.96	0.98	0.85	0.32
Pride	0.20	0.16	0.15	1.02	1.06	0.89	0.15
Joy	0.79	0.89	0.32	1.10	1.15	1.09	0.32
Amusement	0.20	0.16	0.92	1.06	1.05	1.06	0.16
Pleasure	0.33	0.70	0.56	1.07	1.08	0.91	0.33
Relief	0.73	0.26	0.56	1.09	0.93	0.98	0.26
Interest	0.27	0.31	0.56	0.99	0.98	0.95	0.27
Surprise	0.56	0.22	0.20	0.94	0.92	0.83	0.20

Note. NN = nearest-neighbor dissimilarity. Mean dissimilarity represents the average dissimilarity between each emotion and all other emotions within a modality. Minimum NN across modalities represents the smallest nearest-neighbor dissimilarity observed across the audiovisual, visual, and auditory modalities. Larger values indicate emotions that remain relatively distinct across all three modalities.

Cross-modal Cluster Agreement

Table S17

Per-Emotion Cross-Modal Neighbour Retention Across Modality Pairs

Emotion	Mean neighbour Jaccard	Singleton in all modalities
Disgust	—	Yes
Fear	—	Yes
Anger	0.17	No
Pride	0.17	No
Amusement	0.17	No
Interest	0.17	No
Irritation	0.00	No
Sadness	0.00	No
Despair	0.00	No
Anxiety	0.00	No
Joy	0.00	No
Pleasure	0.00	No
Relief	0.00	No
Surprise	0.00	No

Note. Neighbour retention was quantified as the mean Jaccard overlap of each emotion’s same-cluster neighbours across modality pairs. Higher values indicate greater consistency in an emotion’s cluster neighbours across modalities. Emotions that formed singleton clusters in all modalities had no same-cluster neighbours to compare and are therefore indicated by an em dash (—).
